# 2590. Efficacy of Doxycycline Versus Azithromycin in Community Acquired Pneumonia

**DOI:** 10.1093/ofid/ofad500.2205

**Published:** 2023-11-27

**Authors:** Julie Mei, Sharon Blum, Michael Bosco, Meredith Akerman, Andrew Fleming

**Affiliations:** NYU Langone Hospital - Long Island, Brooklyn, New York; NYU Langone Health - Long Island, Lawrence, New York; NYU Langone Hospital - Long Island, Brooklyn, New York; NYU Langone - Long Island, mineola, New York; NYU Langone Hospital - Long Island, Brooklyn, New York

## Abstract

**Background:**

Guideline-based CAP treatment has consisted of 2 preferred regimens: a beta lactam with a macrolide (azithromycin), or monotherapy with fluoroquinolone or beta lactam with doxycycline as an alternative regimen. While doxycycline may represent a more attractive option due to concerns for increased cardiovascular risks associated with azithromycin, individual trials assessing efficacy

**Methods:**

Individuals over 18, admitted for greater than 48 hours at NYU Brooklyn, NYU Long Island, and NYU Tisch from January 1, 2019 to December 31, 2022 with clinical or radiographic evidence of pneumonia treated with ceftriaxone plus either azithromycin or doxycycline were included. Subjects were excluded if received alternative antibiotics for >24 hours, were hospitalization for >48 hours within the last 90 days, were pregnant or breastfeeding, had history of structural lung disease, active lung cancer, suspected tuberculosis, or bloodstream infections, immunocompromising condition, ventilated, or with non-infectious cause of pulmonary infiltrates.

**Results:**

A total of 91 patients met inclusion criteria, 42 patients in the azithromycin group and 49 patients in the doxycycline group. Primary outcomes defined as treatment failure requiring broadening of antibiotics, all-cause inpatient mortality and time to clinical stability were similar between the groups. In the azithromycin group, 11.9% of patients required broadening antibiotics compared to 6.12% in the doxycycline group (p=0.46). Time to clinical stability was 3 in the azithromycin group versus 3.14 days (p=0.6) in the doxycycline group. Secondary outcomes including time to hospital discharge, time to stepdown to oral antibiotics and length of treatment had no difference between groups. There was no difference in safety outcomes between groups.

**Conclusion:**

Azithromycin and doxycycline were comparable in terms of efficacy and safety when treating CAP. These findings can be added to the existing body of evidence supporting doxycycline as an attractive option for atypical CAP coverage.

**Disclosures:**

**All Authors**: No reported disclosures

